# A potential brain functional biomarker distinguishing patients with Crohn’s disease with different disease stages: a resting-state fMRI study

**DOI:** 10.3389/fnins.2024.1361320

**Published:** 2024-03-04

**Authors:** Mengting Huang, Guina Ma, Yan Zou, Hui Ma, Wenliang Fan, Xin Li, Liangru Zhu, Ping Han, Huan Wang, Heshui Shi

**Affiliations:** ^1^Department of Radiology, Union Hospital, Tongji Medical College, Huazhong University of Science and Technology, Wuhan, China; ^2^Hubei Province Key Laboratory of Molecular Imaging, Wuhan, China; ^3^Division of Gastroenterology, Union Hospital, Tongji Medical College, Huazhong University of Science and Technology, Wuhan, China

**Keywords:** Crohn’s disease, amplitude of low frequency fluctuations, resting-state functional magnetic resonance imaging, psychological disorders, brain functional biomarker

## Abstract

**Background:**

The previous studies have demonstrated that patients with Crohn’s disease in remission (CD-R) have abnormal alterations in brain function. However, whether brain function changes in patients with Crohn’s disease in activity (CD-A) and the relationship with CD-R are still unclear. In this study, we aimed to investigate whether the different levels of disease activity may differentially affect the brain function and to find the brain functional biomarker distinguishing patients with different disease stages by measuring the amplitude of low frequency fluctuations (ALFF).

**Methods:**

121 patients with CD and 91 healthy controls (HCs) were recruited. The clinical and psychological assessment of participants were collected. The criteria for the disease activity were the Crohn’s disease activity index (CDAI) scores. CD-R refers to CD patients in remission which the CDAI score is less than 150. Conversely, CD-A refers to CD patients in activity which the CDAI score is ≥150. The ALFF was compared among three groups by performing one-way analysis of variance, followed by a *post hoc* two-sample t-test. Differences among the groups were selected as seeds for functional connectivity analyses. We also investigated the correlation among clinical, psychological scores and ALFF. Binary logistic regression analysis was used to examine the unique contribution of the ALFF characteristics of the disease stages.

**Results:**

There were widespread differences of ALFF values among the 3 groups, which included left frontal pole (FP_L), right supramarginal gyrus (SG_R), left angular gyrus (AG_L), right cingulate gyrus (CG_R), right intracalcarine cortex (IC_R), right parahippocampal gyrus (PG_R), right lingual gyrus (LG_R), right precuneous cortex (PC_R), left occipital fusiform gyrus (OFG_L). Significant brain regions showing the functional connections (FC) increased in FP_L, SG_R, PC_R and OFG_L between CD-A and HCs. The erythrocyte sedimentation rate had a negative correlation with the ALFF values in PC_R in the patients with CD. The phobic anxiety values had a negative correlation with the ALFF values in OFG_L. The psychoticism values had a negative correlation with ALFF values in the IC_R. And the hostility values had a positive correlation with the ALFF values in CG_R. Significant brain regions showing the FC increased in FP_L, SG_R, CG_R, PG_R, LG_R and OFG_L between CD-R and HCs. In binary logistic regression models, the LG_R (beta = 5.138, *p* = 0.031), PC_R (beta = 1.876, *p* = 0.002) and OFG_L (beta = 3.937, *p* = 0.044) was disease stages predictors.

**Conclusion:**

The results indicated the significance of the altered brain activity in the different disease stages of CD. Therefore, these findings present a potential identify neuroimaging-based brain functional biomarker in CD. Additionally, the study provides a better understanding of the pathophysiology of CD.

## 1 Introduction

Crohn’s disease (CD) is a chronic and relapsing gastrointestinal disease. The incidence of CD is increasing globally. Epidemiological studies have shown that the incidence of inflammatory bowel disease in China is the highest in Asia, and the incidence is higher in the south and lower in the north (Yang et al., 2014; Ng et al., 2017). The main symptoms of CD may include fatigue, abdominal pain, diarrhea, weight loss and fever and may range from mild to severe. In addition to gastrointestinal manifestations, patients with CD experience other systemic manifestations and complications, such as arthritis (Roda et al., 2020; Pecci-Lloret et al., 2023).

The disease typically has progressive or alternant course. Inflammatory relapses of CD are unexpected and unpredictable, which might cause intestinal damage or disability in the exacerbation, and it varies among patients. Although significant advances in the clinical management, CD has a significant negative impact on the quality of life of patients. Kochar et al. found that patients with Crohn’s disease in activity (CD-A) had a lower quality of life than patients with Crohn’s disease in remission (CD-R) (Cioffi et al., 2020). Moreover, patients with CD typically require lifelong medication, which seriously affects their quality of life and increases psychological distress. The previous studies have found that disease activity in CD patients is associated with psychological disorders (Huang et al., 2023). Thus, the search for effective biomarker is essential for distinguishing the CD-A and CD-R to implementation of effective interventions.

However, the mechanisms of progressive or alternant of the disease course in CD remain unclear. A better understanding of the alterations in brain activity in patients with CD in different disease states may shed light on the pathophysiology characteristics. Currently, the symptoms of CD are not limited to the gastrointestinal tract, but can also affect other organs, such as the brain (Huang et al., 2022). Recent studies have shown that functional abnormalities of the brain-gut axis (BGA) play a key role in the pathogenesis and development of CD (Al Omran and Aziz, 2014). The BGA are a connection between the brain and gut that is invisible, bi-directional, and composes of the central nervous system, the enteric nervous system, the hypothalamic–pituitary–adrenal (HPA) axis as well as the autonomic nervous system (Gracie et al., 2018). To examine the alterations in brain regions and determine their interactions, neuroimaging is a valuable technique that is non-invasive and is able to have real-time detection such as resting-state functional magnetic resonance imaging (rs-fMRI) based on blood oxygenation level dependent signal.

The oscillation amplitude of spontaneous brain activity has attracted increasing attention. The amplitude of low-frequency fluctuations (ALFF) is recommended as an efficient index for quantifying the regional features of low-frequency oscillations of spontaneous brain activity (Zang et al., 2007; Wang et al., 2019). Oscillation amplitude may represent potentially meaningful and firm baseline brain function in resting states. Static ALFF alterations have been reported in patients with CD; nevertheless, most of these studies have attach importance to the CD-R, and inconsistent findings have been reported (Bao et al., 2018; Li et al., 2021a). None of these studies focused on alterations in CD-A. The diagnosis and evaluation of CD require time-consuming, costly and invasive examinations, such as computed tomography enterography, magnetic resonance enterography or electronic endoscopy. rs-MRI may reveal the functional state of the brain to identify functional abnormalities. Hence, elucidate the role of CD symptoms in regulating brain function and how these changes may affect the neural substrate that interacts between pain, emotional state, and gastrointestinal function in patients with CD, including periods of activity or remission by using ALFF in rs-fMRI.

First, the study aimed to explore the difference of brain activity between the CD-A and CD-R groups, and the specific location of corresponding brain regions by the analysis of the ALFF in the rs-fMRI. Second, a partial correlation analysis was performed between the altered regions and psychological assessment and clinical information in CD to obtain the significance of these brain functions. Third, the study investigated the changes in whole-brain functional connectivity of the identified ALFF regions as a seed in the CD. Finally, the altered ALFF was integrated to get an imaging biomarker for distinguish CD-A and CD-R.

## 2 Materials and methods

### 2.1 Ethics statement

The study was approved by the institutional ethics committee (Protocol Number ICH S016). All participants were informed of the experimental procedure and signed informed consent forms.

### 2.2 Subjects

**Figure 1:**
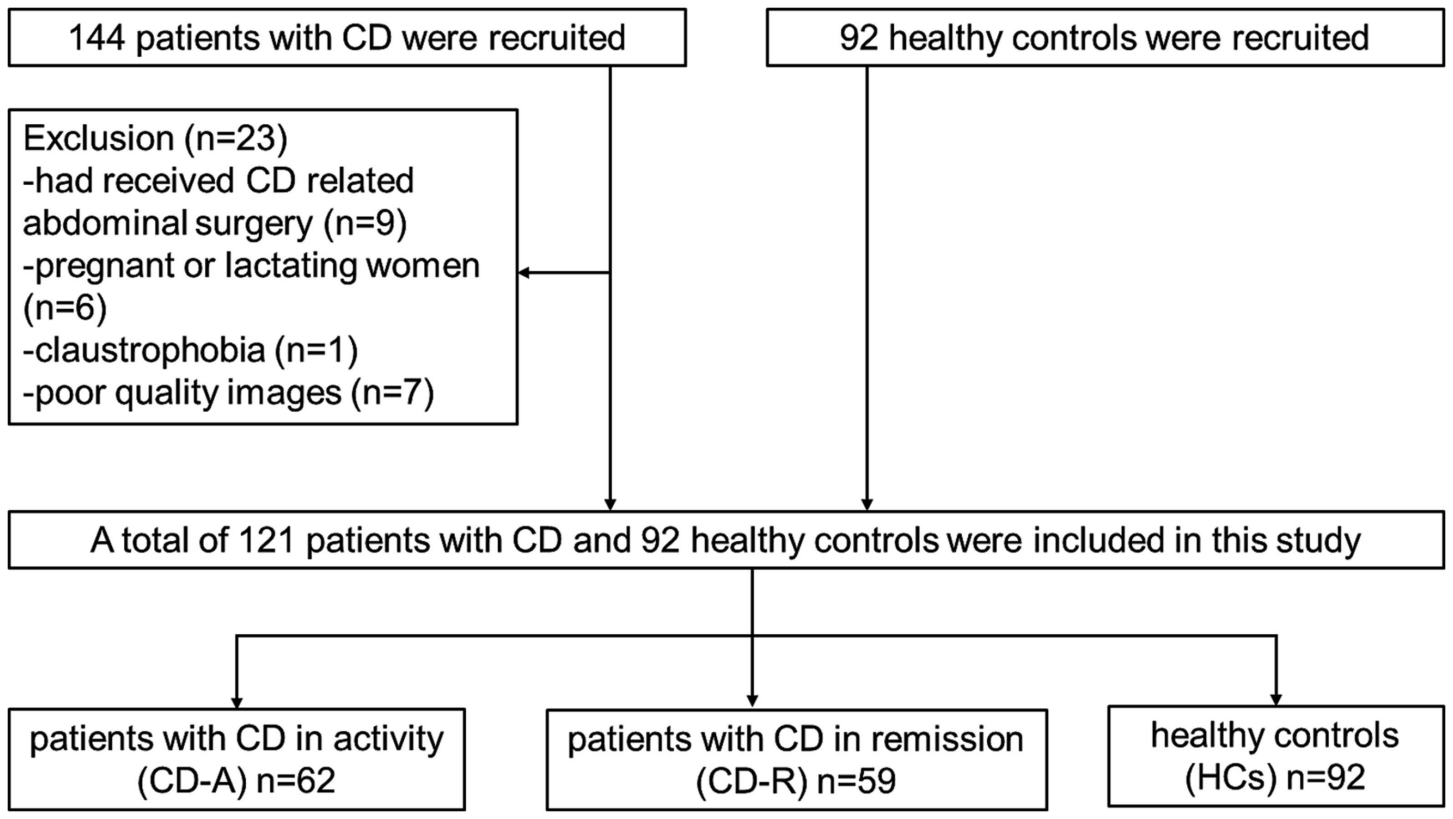
Flow diagram of the enrolled participants. CD, Crohn’s disease.

Initially 236 participants including 144 patients and 92 HCs were recruited from the Department of Gastroenterology of the Union Hospital, Tongji Medical College, Huazhong University of Science and Technology from December 2019 to December 2021. The inclusion criteria of patients with CD were: (1) having a diagnosis of CD confirmed prior to the study by gastroenterologist (Maaser et al., 2019); (2) being over 18 years old; (3) right-handed; (4) native Chinese speaker; and (5) education level of more than 9 years. The inclusion criteria of HCs were: (1) matching with patients by gender and age as much as possible; (2) no disease manifestations of the digestive system; (3) the same with points (2) to (4) on CD. The exclusion criteria of patients and HCs were: (1) receiving CD-relevanted abdominal surgery; (2) with claustrophobia or metal implants; (3) using psychotropic drugs or opioids in the past 3 months; (4) suffering head trauma, tumor or loss of consciousness; (5) pregnant or lactating women; (6) having chronic diseases such as hypertension and diabetes that may affect the structure of the brain; and (7) having poor quality images. The criteria for the disease activity were the Crohn’s disease activity index (CDAI) scores. CD-R refers to CD patients in remission, for which the CDAI score is less than 150. Conversely, CD-A refers to CD patients in activity, in which the CDAI score is ≥150. The flow diagram of the enrolled patients with CD and HCs is shown in Figure 1.

We collected the clinical and psychological features of a clinical database and psychological questionnaire. The questionnaire includes the Inflammatory Bowel Disease Questionnaire (IBDQ) and Symptom Checklist-90 (SCL-90).

### 2.3 MRI data acquisition

All MRI data were acquired on a SIEMENS SKYRA 3.0 T magnetic resonance scanner (Siemens, Germany) equipped with a 32-channel head coil. All of the subjects were instructed to avoid caffeine or other similar substances before examination. Function images were acquired with a single-shot gradient-recalled echo planar imaging sequence parallel acquisition technique GRAPPA acceleration factor 2 using the following parameters: time points were 240; time of repetition (TR) was 2,000 ms; TE was 30 ms; slice thickness was 2.4 mm (no gaps); slices were 60; flip angle (FA) was 90°; field of view (FOV) was 230× 230 mm; matrix size was 96 × 96; voxel size was 2.4 × 2.4 × 2.4 mm; bandwidth was 1796 Hz/Px, and the total scan time was 8′13″.

The T1-weight images were acquired for magnetization prepared rapid gradient echo (MP2RAGE) sequence, parallel acquisition technique GRAPPA acceleration factor 3. The scanner parameters were as follows: slice thickness was 1.0 mm (no gaps); slices were 176; voxel size was 1 × 1 × 1 mm; TR was 5,000 ms; TE was 2.98 ms; FOV was 256× 240 mm; FA was 9°; bandwidth was 240 Hz/Px; and the total scan time was 8′22″. They were kept in the supine position and asked to relax without falling asleep, and keep their eyes closed during scanning. Earplugs and a sponge pad were used to minimize scanner noise to protect hearing and prevent head movement.

### 2.4 MRI data preprocessing

The rs-fMRI data were pre-processed using DPABI software^1^ (V7.0) (Yan et al., 2016) and MATLAB R2021a. The preprocessing steps were as follows: (1) slice timing (disposed the first 10 volumes data); (2) head movement correction (over 2.0 mm translation or over 2.0° rotation in any direction); (3) spatial normalization by using diffeomorphic anatomical registration through exponentiated lie (DARTEL); (4) smoothing by Gaussian kernel of 6 mm full width at half-maximum; and (5) removal of linear trends. In addition, multiple linear regression models were used to regress the effects of white matter and cerebrospinal fluid signals on the processed resting-state data. To reduce the effect of very high frequency-physiological noise or low-frequency drift, the ALFF was computed across 0.01–0.08 Hz, which was the conventional band.

Additionally, based on the ALFF findings, the changed brain regions among the three groups, were chosen as seeds. By using DPABI software, a 6 mm radius around the peak point was taken to encompass the regions of interest (ROIs) for the seed-based functional connections (FC) analysis. The mean time series of the seed regions was extracted for each participant and correlated with each voxel of the whole brain to obtain the seed-based FC maps, which were then transformed to z-maps based on the Fisher z-transformation.

### 2.5 Statistical analysis

One-way analysis of variance (ANOVA), followed by a *post hoc* two-sample *t*-test or Two Independent-sample T-tests were applied to evaluate the normally distributed continuous variables of the clinical and psychological data among groups using IBM SPSS Statistics 26.0 software and GraphPad Prism 8 software. Chi-squared test was used to compare the gender differences. *P* < 0.05 was considered statistically significant.

ANOVA was used to analyze whole brain inter-group differences of ALFF values via DPABI (the statistical module), with the least significant difference (LSD) selected for *post hoc* tests. Gender, age, TIV and FD Jenkinson were the covariates. Then the resultant maps were corrected by using Gaussian random field (GRF) correction (voxel *p* < 0.001, cluster *p* < 0.01). The masks of differential brain regions were created from the results of the ALFF, using the extraction tool (DPABI, Utilities, ROI Signal Extractor), and the functional imaging marker values of brain regions were extracted. Spearman correlations were used to analysis the brain regions and clinical and psychological scores. In addition, the independent sample t-test was used to determine the relationship between disease status and functional imaging marker. Binary logistic regression analysis was used to examine the unique contribution of functional imaging marker on the disease status. The area under curve (AUC) values of the receiver operating characteristic (ROC) curve was used to analyze the ALFF and clinical data in the CD-A and CD-R groups.

## 3 Results

### 3.1 Subjects

Results of the demographic and clinical characteristics among CD-A, CD-R and HCs group are summarized in Table 1. There are no significant differences in gender, age, BMI, education among the participants. Compared to the CD-R, the CD-A has a higher value of C-reactive protein (CRP) (*p* = 0.002), erythrocyte sedimentation rate (ESR) (*p* = 0.0009), platelet levels (*p* = 0.020) and erythrocyte (*p* = 0.004). However, disease duration was not different between CD-A and CD-R groups.

**Table 1:** Clinical and demographics data of patients with Crohn’s disease and healthy controls.

Characteristics	CD-A (*n* = 62)	CD-R (*n* = 59)	HCs (*n* = 92)	*p* value
Sex (Male/Female)	50/12	45/14	66/26	0.446^c^
Age (year)	31.12 ± 12.34	31.00 ± 11.25	28.65 ± 9.49	0.278^a^
Body Mass Index	19.77 ± 3.79	21.01 ± 4.25	19.98 ± 3.37	0.146^a^
Education	16.72 ± 2.31	16.82 ± 3.93	17.40 ± 2.53	0.294^a^
Disease Duration (years)	1.21 ± 1.13	1.50 ± 2.38	-	0.390^b^
C-reactive protein	24.38 ± 30.22	10.86 ± 14.73	-	0.002^b^
Erythrocyte sedimentation rate	21.87 ± 19.35	11.47 ± 13.49	-	0.0009^b^
Platelet levels	299.45 ± 108.62	254.14 ± 102.86	-	0.020^b*^
Crohn’s Disease Activity Index	273.66 ± 35.92	81.35 ± 24.21	-	<0.0001^b^
Erythrocyte	4.49 ± 0.55	5.44 ± 1.96	-	0.0004^b^
Montreal classification	-	-	-	-
L1:L2:L3:L4	16:7:34:5	13:7:35:4	-	0.948^c^
B1:B2:B3:P	15:20:27:31	22:11:24:19	-	0.149^c^
Current therapy	-	-	-	0.001^c^
Nutritional therapy	0	5	-	
5-aminosalicylates	12	24	-	
Biological therapy	24	19	-	
Combined therapy	26	11	-	

CD-A refers to patients with Crohn’s disease in activity. CD-R refers to patients with Crohn’s disease in remission. HCs refer to Healthy controls. L1, ileum; L2, colonic; L3, ileocolonic; L4, upper gastrointestinal tract disease; B1, on inflammatory and no penetrating; B2, inflammatory; B3, penetrating; P, perianal disease. ^a^*p* value for One-way ANOVA. ^b^*p* value for a Two-sample *t*-test. ^c^*p* value for χ2 test.

**Figure 2:**
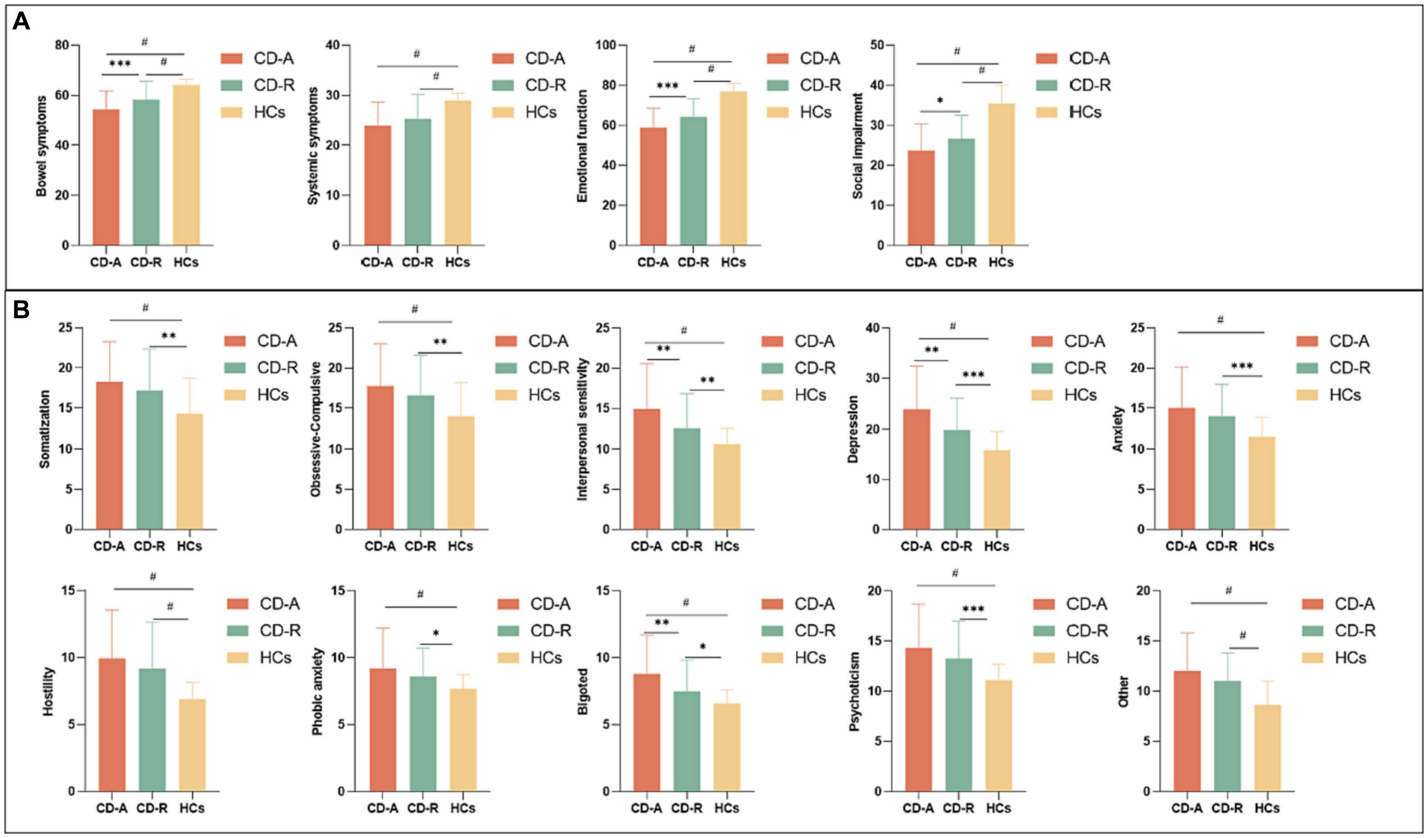
Questionnaire psychological assessment of all participants. **(A)** Comparison of Inflammatory bowel disease questionnaire scores among CD-A, CD-R and HCs group. **(B)** Comparison of Symptom checklist-90 scores among CD-A, CD-R and HCs group. CD-A refers to patients with Crohn’s disease in activity. CD-R refers to patients with Crohn’s disease in remission. HCs refer to Healthy controls. * indicates *p* ≤ 0.05; ** indicates *p* ≤ 0.01; *** indicates *p* ≤ 0.001; # indicates *p* ≤ 0.0001.

The bowel symptoms scores (*p* = 0.0008), emotional function scores (*p* = 0.0005) and social impairment scores (*p* = 0.0107) of IBDQ, interpersonal sensitivity scores (*p* = 0.0051), depression scores (*p* = 0.0017), and bigoted scores (*p* = 0.0021) of SCL-90 of CD-A group were significantly different with CD-R group. A detailed psychological characteristics description among three groups were reported in Figure 2.

### 3.2 Difference in ALFF values among CD-A, CD-R and HCs group

**Figure 3:**
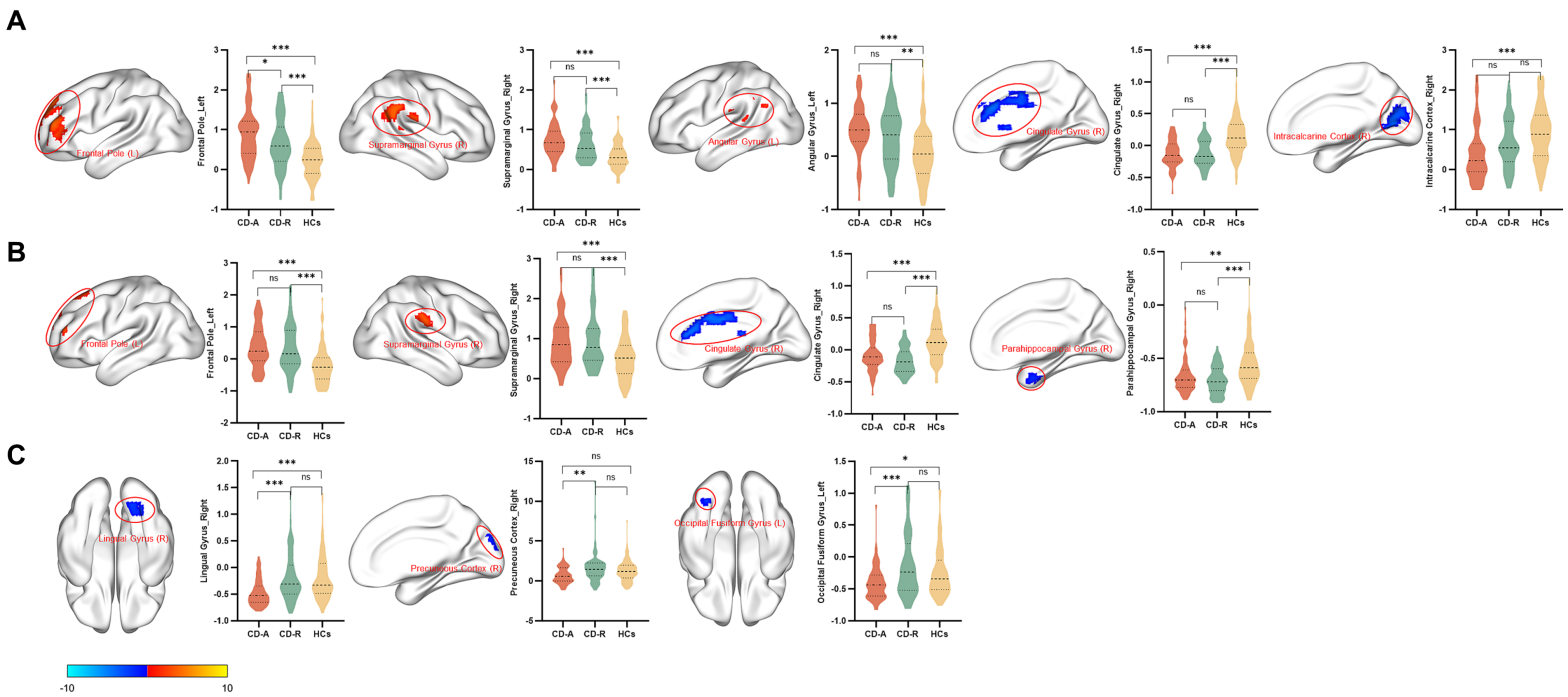
Brain regions with significant differences in amplitude of low frequency fluctuations between patients with Crohn’s disease and healthy controls. The results employed gender, age, and intracranial volume as covariates. Significant increased (red) and decreased (blue) (GRF corrected, voxel *p* < 0.001, cluster *p* < 0.01). The color bar represents the *t*-value. **(A)** Brain regions with significant differences in amplitude of low frequency fluctuations (ALFF) between patients with Crohn’s disease in activity (CD-A) and healthy controls (HCs). Compared with the HCs, increased ALFF values in the left frontal pole, right supramarginal gyrus and left angular gyrus were observed, decreased ALFF values in the right cingulate gyrus and right intracalcarine cortex were observed in the CD-A. **(B)** Brain regions with significant differences in amplitude of low frequency fluctuations between patients with Crohn’s disease in remission (CD-R) and HCs. Increased ALFF values in the left frontal pole and right supramarginal gyrus were observed, decreased ALFF values in the right cingulate gyrus and right parahippocampal gyrus were observed in the CD-R. **(C)** Brain regions with significant differences in amplitude of low frequency fluctuations between CD-A and CD-R. The brain activity of ALFF in the CD-A in the right lingual gyrus, right precuneous cortex, and left occipital fusiform gyrus were lower than the CD-R.

Compared to the HCs, the CD-A group showed higher ALFF values in left frontal pole (FP_L), right supramarginal gyrus (SG_R) and left angular gyrus (AG_L), and showed lower ALFF values in right cingulate gyrus (CG_R) and right intracalcarine cortex (IC_R) (Figure 3A). Compared to the HCs, the CD-R group showed higher ALFF values in FP_L and SG_R, and showed lower ALFF values in CG_R and right parahippocampal gyrus (PG_R) (Figure 3B). The brain activity of ALFF in the CD-A in the right lingual gyrus (LG_R), right precuneous cortex (PC_R), and left occipital fusiform gyrus (OFG_L) was lower than the CD-R (Figure 3C). A detailed description of brain regions with significant differences among CD-A, CD-R and HCs were shown in Table 2. The abbreviation of brain regions based on Harvard-Oxford Cortical Structural Atlas.

**Table 2:** Brain regions with significant differences in amplitude of low frequency fluctuations between patients with Crohn’s disease and healthy controls.

Regions	BA	Hem	MNI peak coordinate			Voxel size	*T* value
			X	Y	Z		
CD-A > HCs	-	-	-	-	-	-	-
Frontal Pole	10	L	−15	69	6	594	4.621
Supramarginal Gyrus	48	R	59	−23	26	56	4.877
Angular Gyrus	22	L	−63	−57	21	45	4.432
CD-A < HCs	-	-	-	-	-	-	-
Cingulate Gyrus	24	R	6	24	24	103	−4.387
Intracalcarine Cortex	17	R	15	−69	12	69	−4.278
CD-R > HCs	-	-	-	-	-	-	-
Frontal Pole	9	L	12	54	37	188	3.842
Supramarginal Gyrus	2	R	66	−24	30	56	3.682
CD-R < HCs	-	-	-	-	-	-	-
Cingulate Gyrus	24	R	3	7	33	165	−4.279
Parahippocampal Gyrus	28	R	24	−6	−39	40	−4.077
CD-A < CD-R	-	-	-	-	-	-	-
Lingual Gyrus	19	R	21	−63	−12	52	−3.203
Precuneous Cortex	19	R	3	−81	42	32	−3.025
Occipital Fusiform Gyrus	19	L	−33	−69	−18	27	−3.260

The results employed gender, age intracranial volume and FD Jenkinson as covariates. The statistical threshold was set at voxel *p* < 0.001, cluster *p* < 0.01, and two-tailed (GRF correction). CD-A refers to Crohn’s disease patients in activity. CD-A refers to patients with Crohn’s disease in activity. CD-R refers to patients with Crohn’s disease in remission. HCs refer to Healthy controls. GMV, gray matter volume; Hem, hemisphere; BA, Brodmann area; L, left; R, right.

### 3.3 Difference in FC among the CD-A, CD-R and HCs group

**Figure 4:**
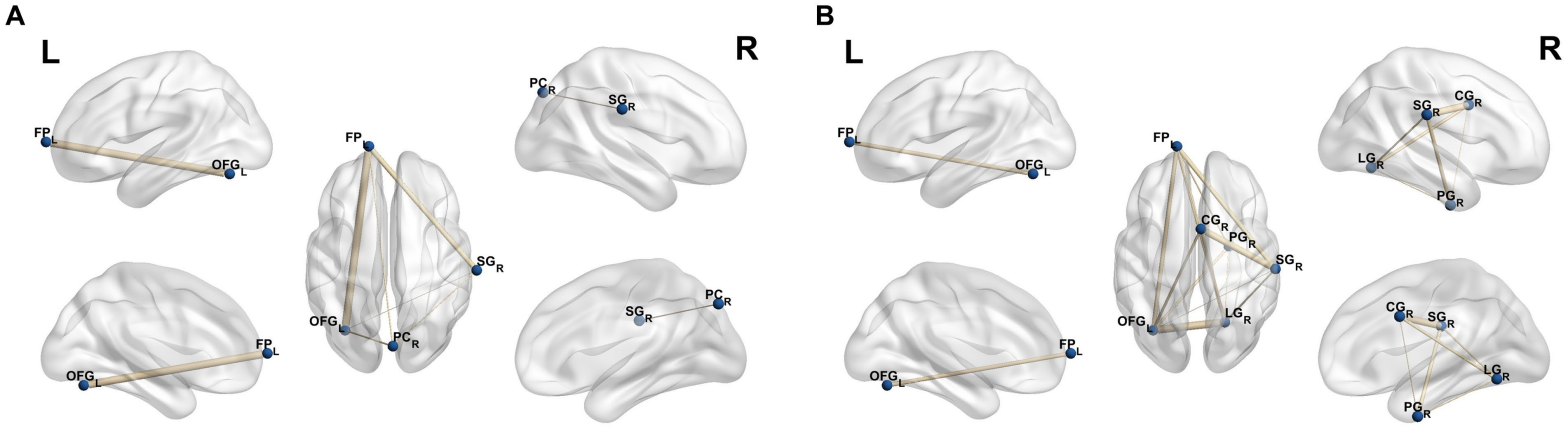
Results of FC analysis selecting the mask of amplitude of low frequency fluctuations as seed point. **(A)** Significant brain regions showing increased functional connections of the FP_L, SG_R, PC_R, OFG_L between patients with Crohn’s disease in activity (CD-A) and healthy controls (HCs) (*p* < 0.05, FEW correction); **(B)** Significant brain regions showing increased functional connections of the FP_L, SG_R, CG_R, PG_R, LG_R, OFG_L between patients with Crohn’s disease in remission (CD-R) and HCs (*p* < 0.05, FEW correction). FP_L, Frontal Pole_Left; SG_R, Supramarginal Gyrus_Right; PC_R, Precuneous Cortex_Right; OFG_L, Occipital Fusiform Gyrus_Left; CG_R, Cingulate Gyrus_Right; PG_R, Parahippocampal Gyrus_Right; LG_R, Lingual Gyrus_Right.

As showed in Figure 4A, based on the ALFF result, the strengths of the FC in the FP_L, SG_R, PC_R, OFG_L was found to be increased in CD-A compared with the HCs. Compare with the HCs, the strengths of the FC in the FP_L, SG_R, CG_R, PG_R, LG_R, OFG_L was observed to be increased in the CD-R, which were shown in Figure 4B.

### 3.4 Correlation among clinical data, psychological data, and ALFF values in patients with CD

**Figure 5:**
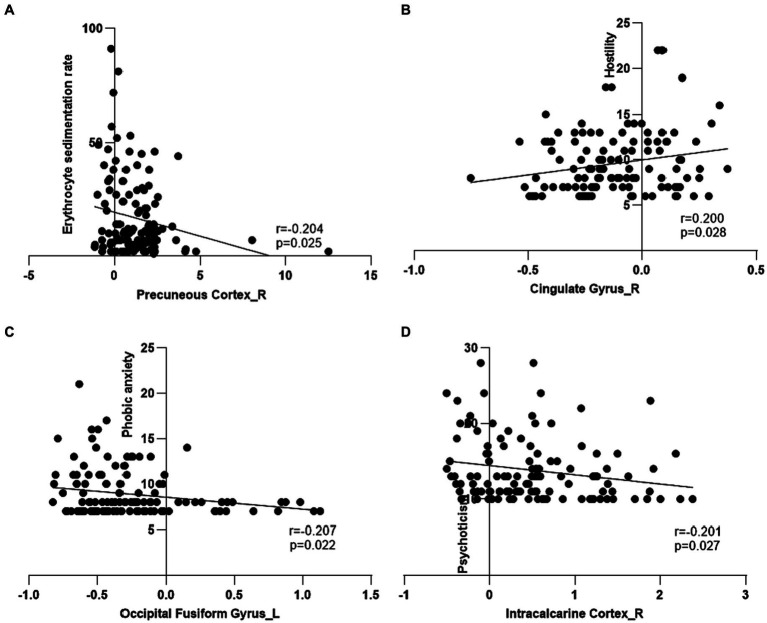
Correlation among clinical features, psychological assessment scores and amplitude of low frequency fluctuations of regional brain in patients with Crohn’s disease. **(A)** The amplitude of low frequency fluctuations (ALFF) in the right precuneous cortex had a negative correlation with erythrocyte sedimentation rate in the patients with Crohn’s disease (CD). **(B)** The ALFF in the right cingulate gyrus had a positive correlation with hostility in the patients with CD. **(C)** The ALFF in the left occipital fusiform gyrus had a negative correlation with phobic anxiety in the patients with CD. **(D)** The ALFF in the right intracalcarine cortex had a negative correlation with psychoticism in the patients with CD.

The erythrocyte sedimentation rate had a negative correlation with the ALFF values in PC_R in the patients with CD (Figure 5A). The phobic anxiety values had a negative correlation with the ALFF values in OFG_L (Figure 5C). The psychoticism values had a negative correlation with ALFF values in the IC_R (Figure 5D). And the hostility values had a positive correlation with the ALFF values in CG_R (Figure 5B). The results for subgroups shown in Supplementary Figure S1. In this study, there were no significant correlations between the ALFF values of aberrant regions and disease duration in patients with CD.

### 3.5 Independent predictors of the disease status

**Figure 6:**
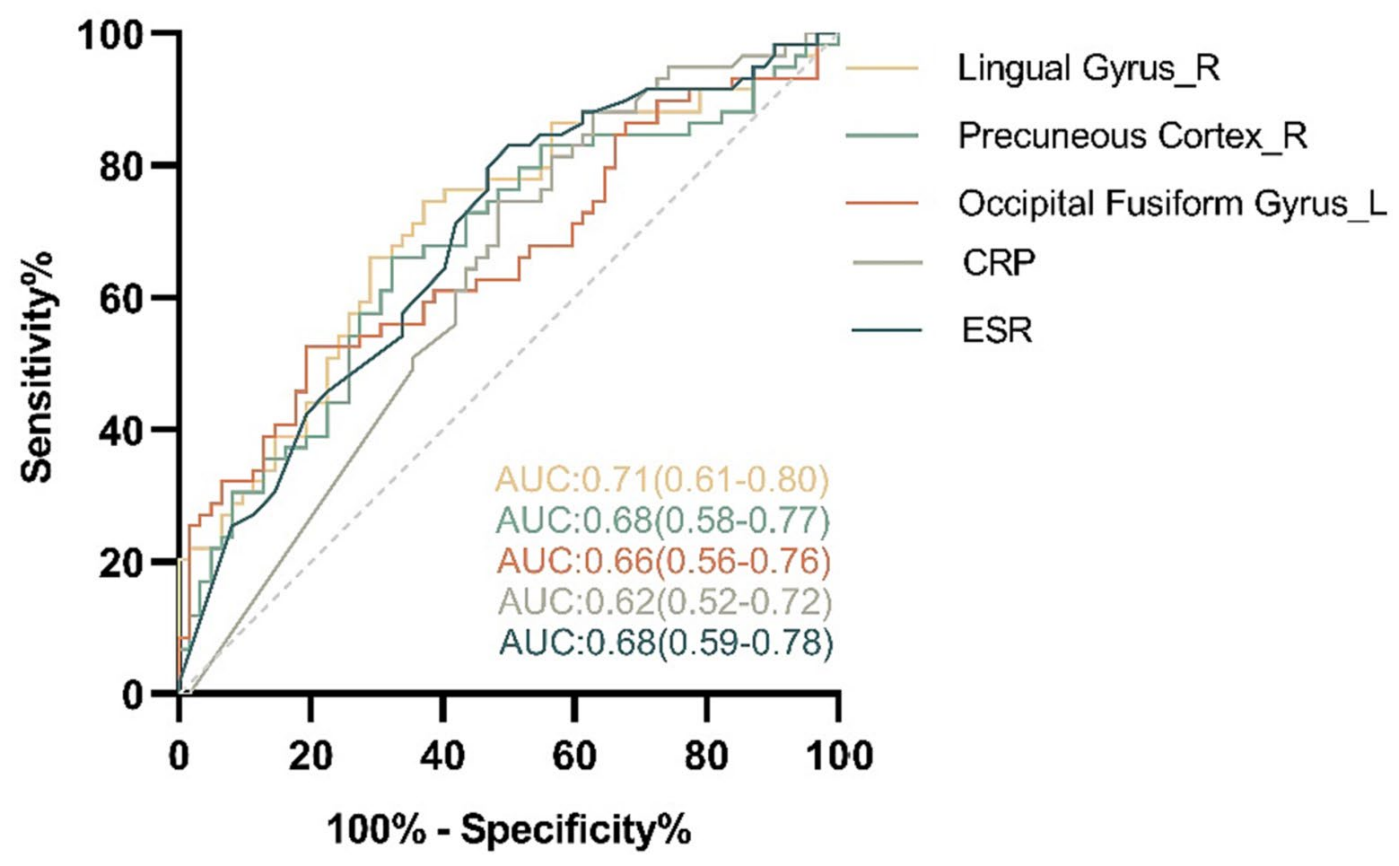
The area under curve (AUC) values of the receiver operating characteristic (ROC) analysis between the amplitude of low frequency fluctuations of regional brain and clinical features in patients with Crohn’s disease. In the differentiation of the patients with Crohn’s disease in activity (CD-A) and the patients with Crohn’s disease in remission (CD-R), the amplitude of low frequency fluctuations parameters of Lingual Gyrus_R (AUC = 0.71, *p* < 0.0001) had the highest accuracy, followed by the Precuneous Cortex_R (AUC = 0.68, *p* = 0.0009), followed by the Occipital Fusiform Gyrus_L (AUC = 0.66, *p* = 0.0023) and ESR (AUC = 0.68, *p* = 0.0004), and the CRP (AUC = 0.62, *p* = 0.0184) had the lowest accuracy. R, right; L, left; CRP, C-reactive protein; ESR, Erythrocyte sedimentation rate.

Univariate analysis suggested that the ALFF value in FP_L (*p* = 0.036), IC_R (*p* = 0.042), LG_R (*p* < 0.001), PC_R (*p* = 0.002) and OFG_L (*p* = 0.001) was statistically significant and were included in the subsequent binary logistic regression analysis. Binary logistic regression analyses showed that the LG_R (beta = 5.138, *p* = 0.031), PC_R (beta = 1.876, *p* = 0.002), and OFG_L (beta = 3.937, *p* = 0.044) was predictors of disease status, which was shown in Table 3. In the differentiation of CD-A and CD-R, the ALFF parameters of LG_R (AUC = 0.71, *p* < 0.0001) had the highest accuracy, followed by the PC_R (AUC = 0.68, *p* = 0.0009), followed by the OFG_L (AUC = 0.66, *p* = 0.0023) and ESR (AUC = 0.68, *p* = 0.0004), and the CRP (AUC = 0.62, *p* = 0.0184) had the lowest accuracy (Figure 6).

**Table 3:** Results of the analysis of binary logistic regression analysis on disease stages.

The factors	Hosmer and Lemeshow test	S.E.	Wald	Beta	95% CI	*p* value
Univariate		-		-	-	-
Frontal Pole_L	0.141	0.301	4.374	0.533	0.296–0.961	0.036
Supramarginal Gyrus_R	0.449	0.449	2.420	0.497	0.206–1.199	0.120
Cingulate Gyrus_R	0.796	0.848	0.011	1.095	0.208–5.764	0.915
Angular Gyrus_L	0.232	0.360	2.353	0.576	0.284–1.166	0.125
Intracalcarine Cortex_R	0.536	0.283	4.151	1.781	1.022–3.102	0.042
Frontal Pole_L	0.691	0.260	0.068	0.935	0.561–1.557	0.795
Cingulate Gyrus_R	0.522	0.823	3.051	0.238	0.047–1.192	0.081
Supramarginal Gyrus_R	0.828	0.279	0.184	1.127	0.653–1.946	0.668
Parahippocampal Gyrus_R	0.576	1.250	2.625	0.132	0.011–1.529	0.105
Lingual Gyrus_R	0.190	0.673	13.577	11.925	3.191–44.572	<0.001
Precuneous Cortex_R	0.362	0.173	9.458	1.702	1.213–2.388	0.002
Occipital Fusiform Gyrus_L	0.747	0.570	10.620	6.412	2.097–19.606	0.001
Multivariate	-	-	-	-	-	-
Lingual Gyrus_R	0.345	0.759	4.653	5.138	1.161–22.730	0.031
Precuneous Cortex_R		0.205	9.451	1.876	1.256–2.801	0.002
Occipital Fusiform Gyrus_L		0.681	4.056	3.937	1.037–14.944	0.044

SE, standardized error.

## 4 Discussion

Chronic and inflammatory bowel involvement makes CD an ideal candidate to explore the GBA hypothesis. Currently, a growing number of studies have begun to explore the extent of brain alterations in patients with CD. As brain changes in CD may be an effect of multiple underlying mechanisms that are still poorly understood, specific differences between two disease activity state are not easily explained. Given that CD symptoms that lead to psychological disorders are affected differently bydisease activity state, we stratified CD patients according to the level of disease activity in this study. This study investigated potential brain functional alterations among the CD-A, CD-R and HCs groups by the ALFF of rs-fMRI. Several brain regions, particular parts of the lingual gyrus, precuneous cortex and occipital fusiform gyrus, showed alterations in the brain function associated with different levels of disease activity in patients with CD. We also found that the CD-A and CD-R group shown increased FC in multiple brain regions compared with HCs. In addition, we also found that the altered of ALFF values in right lingual gyrus, right precuneous cortex, and left occipital fusiform gyrus might potentially serve as brain functional biomarker for distinguishing patients with CD in different disease stages.

The results of this study shown decreased ALFF values in the right lingual gyrus, right precuneous cortex, and left occipital fusiform gyrus in CD-A compared with CD-R. Meanwhile, the ALFF values in the right precuneous cortex had a negative correlation with erythrocyte sedimentation rate in the patients with CD, and the ALFF values in the left occipital fusiform gyrus had a negative correlation with phobic anxiety in the patients with CD. These differences of ALFF values in these brain regions are independent of HCs. In the hierarchical multiple regression analysis of this study, the ALFF values of these brain regions are independent predictors of disease activity. It may reflect variability associated to different stages of disease activity state and to differing levels of clinical or psychological symptom intensity. And the altered of ALFF might potentially serve as brain functional biomarker for distinguishing patients with CD in different disease activity state.

The medial occipital lobe is a region of the cerebral cortex composed of the lingual gyrus and cuneus. It is necessary for both basic and higher levels visual processing. The medial occipital lobe is the center of many long-range association fibers that holds the cuneus and lingual gyrus and facilitates visual processing (Palejwala et al., 2021). The precuneus play a central role in in a wide spectrum of highly integrated tasks, such as episodic memory, visuo-spatial imagery, and self-processing operations (Cavanna and Trimble, 2006). In addition, the precuneus is involved in the interweaving network associated with the self-conscious nerve. Higher levels of circulating cytokines could cross the blood–brain barrier, from a gut to brain perspective, inducing oligodendrocyte, astrocyte apoptosis and neuroinflammation, which results in multiple kinds of abnormalities in the brain. Together, abnormal activities in these brain regions may suggest abnormal brain functions related to the regulation of visceral sensation in patients with CD.

On the other hand, compared with HCs, the altered of ALFF values in frontal pole, supramarginal gyrus, and cingulate gyrus was detected across the CD-A and CD-R group. These brain regions were the same as the results of Li et al. (2021a). It is also noteworthy that the ALFF in the right cingulate gyrus had a positive correlation with hostility in the patients with CD in this study. This altered neurological profile was reported in patients with affective disorders or anxiety and depression (Rive et al., 2013; Qi et al., 2023; Yu et al., 2023). The frontal pole receives input from the limbic system, and plays a key role in emotional memory storage and emotional regulation (Bermpohl et al., 2006; Bramson et al., 2020). Studies have shown that the frontal lobe is crucial to the development of emotional intelligence in children (Rosso et al., 2004). The anterior midcingulate cortex had a significant role in feedback-mediated decision making (Vogt, 2013). Previous studies have found that the gray matter volume in is reduced in patients with CD (Kong et al., 2021). The anterior cingulate had impaired functions in patients with depression. This suggests that these overlapping brain regions presumably is an extremely interconnected system, linked collaboratively to regulation of emotions. This possible synergistic effect was further confirmed in the subsequent FC results.

Multiple FC alterations in patients with CD were found. First, increased FC in the left frontal pole, right supramarginal gyrus, right cingulate gyrus, right parahippocampal gyrus, right lingual gyrus, and left occipital fusiform gyrus were found when compared with CD-R and HCs. Interestingly, these results, with the exception of frontal pole and cingulate gyrus, have not been reported in previous studies. This may be due to the fact that the sample size of this study was the largest of any published articles on rs-fMRI in patients with CD. The function of these brain regions is important for cognitive and emotional regulation (Li et al., 2021b; Yue et al., 2023; Zhao et al., 2023). Our neuroimaging findings may provide a neurophysiological substrate that may potentially explain the symptoms of psychological impairment observed in patients with CD, beyond the inflammatory stage.

Moreover, decreased connectivity in CD-A relative to HCs was also found in left frontal pole, right supramarginal gyrus, right precuneous cortex and left occipital fusiform gyrus. This is particularly important result that above are involved in the regulation of emotional, cognitive, and visual centers, and has not been reported in previous study. Interestingly, in this study we did not find the altered of FC between CD-A and CD-R. This may be due to the crossover of disease course between CD-A and CD-R group. It may indicate the potential effect of disease course on the FC of patients in CD. Future studies will need to be designed to test this hypothesis. Taken together, our results suggest that the FC might be a potentially useful target in CD to evaluate the contribution of disease activity in modulating brain response.

Whereas the ALFF values in the left angular gyrus, right intracalcarine cortex altered when the CD-A was compared with HCs, these changes were not seen in the CD-R group. Similarly, when the CD-R was compared with HCs, the ALFF value in the right parahippocampal gyrus changes, but it did not appear in the CD-A group. This probably due to the number of studies reporting rs-fMRI data. The angular gyrus located primarily in the anterolateral area of the parietal lobe, is associated with a spectrum of higher order cognitive functions, such as transmitting visual information to the Wernicke area as well as various languages, cognitive functions (Rockland, 2023). The intracalcarine cortex, located in the occipital lobe, was the primary visual cortex center, and transmits information from the retina. The parahippocampal gyrus, a part of the cerebral cortex, plays a vital role in high cognitive functions including retrieval and visuospatial processing and memory encoding, which surrounds the hippocampus (van Strien et al., 2009; Lin et al., 2021). The BGA is a connection between the gut and the brain that is bi-directional. By changing the state of the HPA axis, the autonomic nervous system directly affects the modulation of gut functions. In turn, the input information of the gut and mental stress can activate related brain regions. Meanwhile, enteric microbiota is also likely to interact with nervous system via neural signaling, endocrine and immune mechanisms.

Although the study supplemented reports from the past, the sample size is also the largest reported in the literature so far. It had a number of limitations. First, the study was a single-center cross-sectional study in which all participants were included from a hospital. Second, the cross-sectional nature of the data precludes us from concluding the causality of the relationships between the altered of ALFF values and the disease activity. In the future, longitudinal research should be conducted to confirm the causality.

## 5 Conclusion

This study demonstrated obvious differentially alterations in ALFF brain activity and FC in patients with CD in the different stages of disease activity, which may potentially reflect a putative pathway influenced by persistent inflammatory activity. In addition, the alteration of ALFF was associated with psychological impairment, reflecting brain nerve dysfunction in CD. Finally, the altered ALFF in LG_R, PC_R, and OFG_L proved to be a powerful tool in differentiating the disease stages of CD and is therefore a potential neuroimaging biomarker.

## Data availability statement

The raw data supporting the conclusions of this article will be made available by the authors, without undue reservation.

## Ethics statement

The studies involving humans were approved by Institutional Ethics Committee of Tongji Medical College of Huazhong University of Science and Technology. The studies were conducted in accordance with the local legislation and institutional requirements. The participants provided their written informed consent to participate in this study.

## Author contributions

MH: Conceptualization, Data curation, Formal analysis, Investigation, Methodology, Project administration, Software, Supervision, Validation, Visualization, Writing – original draft, Writing – review & editing. GM: Data curation, Investigation, Project administration, Supervision, Writing – original draft, Writing – review & editing. YZ: Formal analysis, Investigation, Methodology, Project administration, Software, Writing – review & editing. HM: Conceptualization, Data curation, Formal analysis, Investigation, Methodology, Project administration, Software, Supervision, Validation, Writing – review & editing. WF: Data curation, Formal analysis, Methodology, Project administration, Supervision, Validation, Visualization, Writing – review & editing. XL: Conceptualization, Data curation, Investigation, Methodology, Software, Supervision, Writing – review & editing. LZ: Formal analysis, Funding acquisition, Project administration, Resources, Supervision, Visualization, Writing – review & editing. PH: Conceptualization, Data curation, Formal analysis, Investigation, Methodology, Supervision, Writing – review & editing. HW: Conceptualization, Data curation, Formal analysis, Funding acquisition, Investigation, Methodology, Project administration, Resources, Software, Supervision, Validation, Visualization, Writing – review & editing. HS: Conceptualization, Data curation, Formal analysis, Investigation, Methodology, Project administration, Resources, Software, Supervision, Validation, Visualization, Writing – review & editing.

## Footnotes

^1^ http://rfmri.org/dpabi
