# Supplementary material for: A potential brain functional biomarker distinguishing patients with Crohn’s disease with different disease stages: a resting-state fMRI study
Source: Front Neurosci. 2024 Mar 4;18:1361320. doi: 10.3389/fnins.2024.1361320 (PMC10945013; doi:10.3389/fnins.2024.1361320)
Supplement: Supplementary file 1 [file Image_1.pdf]

Supplementary Material

1 Supplementary Figures and Tables

1.1 Supplementary Figure

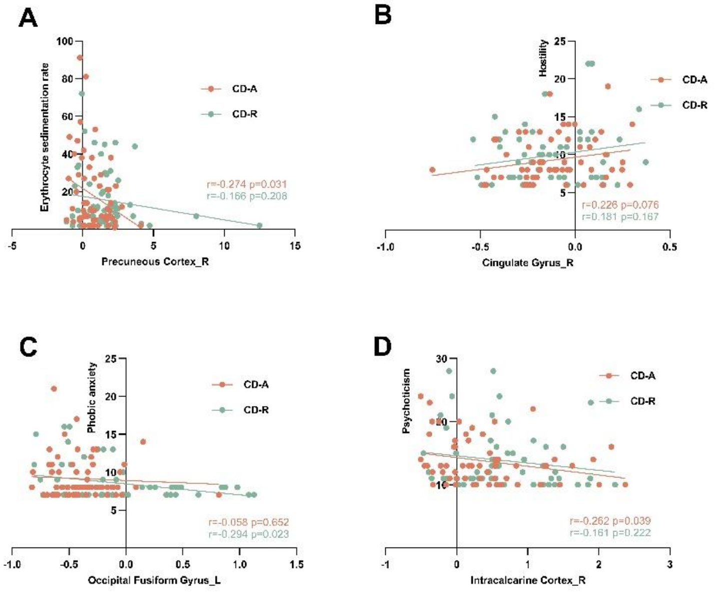

Figure S1. Correlation among clinical features, psychological assessment scores and amplitude of low frequency fluctuations of regional brain in CD-A group and CD-R group. Note: Abbreviations: CD-A: patients with Crohn's disease in activity, CD-R: patients with Crohn's disease in remission, R: right, L: left.
